# Causality between rheumatic diseases and iron-deficiency anemia: A 2-sample, 2-step mediation Mendelian randomization investigation

**DOI:** 10.1097/MD.0000000000046345

**Published:** 2026-05-12

**Authors:** Wei Huang, Luyao Lv, Yaqi Zhang, Tianyu Jin, Yifan Cheng, Linyu Geng, Xuebing Feng

**Affiliations:** a Department of Rheumatology and Immunology, Drum Tower Clinical Medical College, Nanjing Medical University, Nanjing, Jiangsu, China; b Department of Rheumatology and Immunology, Affiliated Hospital of Medical School, Nanjing Drum Tower Hospital, Nanjing University, Nanjing, Jiangsu, China; c Department of Rheumatology and Immunology, Drum Tower Clinical Medical College, Nanjing University of Chinese Medicine, Nanjing, Jiangsu, China; d Department of Rehabilitation Medicine, the Second Affiliated Hospital and Yuying Children’s Hospital of Wenzhou Medical University, Wenzhou, Zhejiang, China; e Center for Rehabilitation Medicine, Department of Neurology, Zhejiang Provincial People’s Hospital, Affiliated People’s Hospital, Hangzhou Medical College, Hangzhou, Zhejiang, China.

**Keywords:** iron-deficiency anemia, iron status, Mendelian randomization, rheumatic diseases, single-nucleotide polymorphisms

## Abstract

Growing evidence suggests that patients with rheumatic disease are associated with iron-deficiency anemia (IDA). However, the causal relationship between rheumatic disease and IDA risk remains unclear. To investigate this, we conducted a Mendelian randomization (MR) study using genetic variants from the large genome-wide association studies. In this study, we primarily investigated common rheumatic diseases, which include rheumatoid arthritis (RA), systemic lupus erythematosus (SLE), Sjogren syndrome (SS), systemic sclerosis (SSc) as exposures, with IDA as the outcome and iron status as the mediator. We extracted significant and independent single-nucleotide polymorphisms from the large European genome-wide association studies for each trait: RA (5427 cases and 479,171 controls), SLE (5201 cases and 9066 controls), SS (1290 cases and 213,145 controls) and SSc (302 cases and 213,145 controls). Outcomes comprised IDA (2941 cases and 481,657 controls) and 4 iron status biomarkers (serum iron, ferritin, transferrin, transferrin saturation; n = 48,972) to serve as instrumental variables. In the MR analysis, we primarily used the inverse-variance weighting method, supplemented by weighted-median and MR-Egger methods. Additionally, a series of sensitivity analyses were conducted to test the stability of the MR analysis. We identified 10 single-nucleotide polymorphisms for RA, 41 for SLE, 3 for SS, and 7 for SSc as instrumental variables. Univariate bidirectional MR analysis suggests that RA increases IDA risk (OR = 1.058, 95% CI: 1.024–1.094, *P* <.01). However, we found no significant genetic effect for SLE, SS, or SSc. In 2-step MR analysis, multivariate MR (MVMR) indicates that RA and ferritin independently affect IDA risk (RA: OR = 1.073, 95% CI: 1.005–1.146, *P* = .03; ferritin: OR = 0.997, 95% CI: 0.995–0.999, *P* <.01). Additionally, the mediation MR analysis suggested that ferritin partially mediated this effect. Our findings initially provide strong genetic evidence for the association between RA and an increased risk of IDA, with ferritin partially mediating this effect. However, no such association was found between SLE, SS, SSc and IDA. These results could inform the development of preventive strategies and interventions for rheumatic diseases and IDA.

## 1. Introduction

Rheumatic diseases constitute a heterogeneous group of chronic autoimmune and inflammatory disorders that primarily target connective tissues, joints, and occasionally internal organs. Conditions such as rheumatoid arthritis (RA), systemic lupus erythematosus (SLE), Sjögren’s syndrome (SS), and systemic sclerosis (SSc) exhibit a complex immunopathology characterized by persistent inflammation and systemic involvement, often leading to a spectrum of clinical manifestations, from mild symptoms to severe complications that profoundly impact patients’ lives.^[1]^ Within this diverse array of conditions, the systemic nature of rheumatic diseases is underscored by their ability to affect multiple bodily systems, including the hematologic system and iron metabolism.^[2]^ This systemic involvement prompts consideration of potential connections between rheumatic diseases and disruptions in iron metabolism, such as iron-deficiency anemia (IDA).

IDA is a prevalent form of anemia globally, arising from insufficient iron levels in the body to meet physiological demands. It may result from factors such as inadequate dietary iron intake, impaired iron absorption, blood loss, or increased iron requirements.^[3]^ Despite its prevalence, the specific mechanisms underlying IDA development within the context of rheumatic diseases remain incompletely understood.

Chronic inflammation, a hallmark feature of rheumatic diseases, emerges as a significant contributor to IDA. This inflammation disrupts iron distribution within the body, leading to abnormal iron storage in tissues, thereby impeding its effective utilization and potentially triggering anemia.^[4]^ Moreover, specific rheumatic diseases, such as RA, may exacerbate the risk of IDA through factors such as impaired intestinal iron absorption and medication side effects, further predisposing individuals to anemia.^[5]^ Furthermore, shared immunopathological mechanisms between rheumatic diseases and anemia, associated with abnormal activation of the immune system, may indirectly influence iron metabolism and utilization, affecting iron homeostasis and contributing to the development of IDA.^[6]^ Despite the potential mechanisms proposed, the precise relationship between rheumatic diseases and IDA remains elusive, and a definitive causal connection has yet to be conclusively established.

Mendelian randomization (MR) uses inherited genetic variants as instrumental variables (IVs) to estimate causal effects while minimizing confounding and reverse causation, offering a robust alternative when randomized trials are impractical.^[7,8]^ In this study we applied bidirectional univariate MR and 2‑step mediation MR to test whether RA causally elevates the risk of IDA and to determine whether ferritin mediates this link, providing mechanistic insight that may inform targeted management of this common comorbidity.

## 2. Methods

### 2.1. Study design

**Figure 1.**
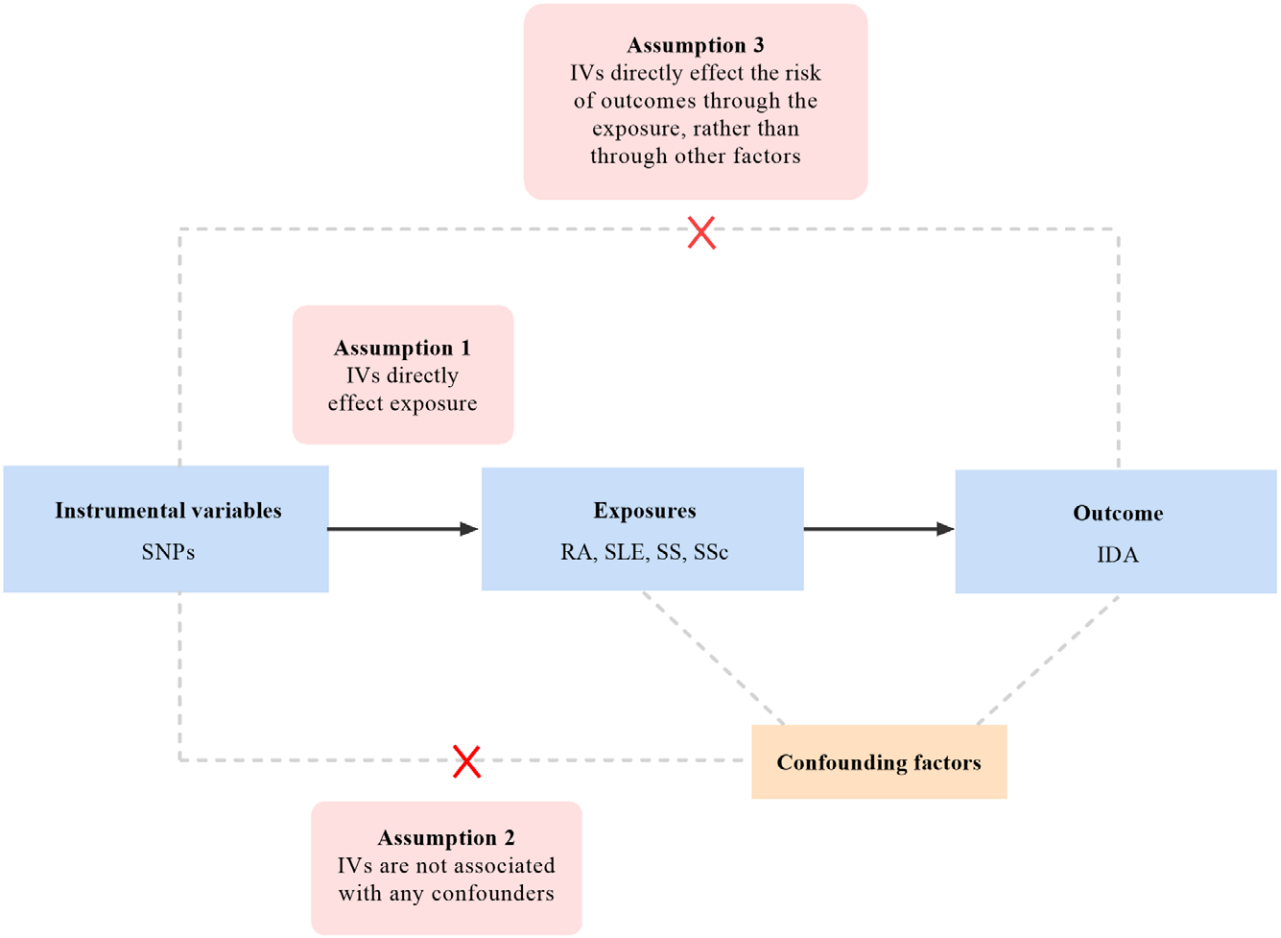
Overview of study design and key assumptions in MR analysis. MR = Mendelian randomization.

Our study was conducted in 2 stages. Initially, we performed a bidirectional UVMR analysis to determine the exposure and the outcome, guided by 3 core MR assumptions, as shown in Figure 1^[9]^: the genetic IVs are significantly associated with the exposure; the genetic IVs are independent of confounders that might affect the outcome; and the genetic IVs influence the outcome only through their effect on the exposure and not via other pathways. Subsequently, a 2-step mediation MR analysis was employed to investigate whether iron status mediated the causal association between the exposure and the outcome.^[10]^

### 2.2. Data source

For RA, we used the most comprehensive GWAS summary statistics available for the European population, which comprised 5427 cases and 479,171 controls.^[11]^ The GWAS for SLE included a cohort of 14,267 individuals, consisting of 5201 cases and 9066 controls, also from the European demographic.^[12]^ For SS, the study dataset included 1290 cases and 213,145 controls within the same population. In the case of SSc, the GWAS data encompassed 302 cases and 213,145 controls, similarly drawn from the European population (https://www.finngen.fi/en). For IDA, the GWAS comprised 484,598 individuals of European descent, with 2941 cases and 481,657 controls.^[13]^ The diagnosis of RA and IDA was determined based on the ICD-10 criteria. For iron status (iron, ferritin, transferrin, and transferrin saturation), we sourced data from the GWAS of the Genetics of Iron Status Consortium. This GWAS involved 48,972 individuals of European descent from 19 cohorts.^[14]^

### 2.3. Selection of genetic instrumental variables

We applied stringent selection criteria to the IVs: the IVs required single-nucleotide polymorphisms (SNPs) that met the genome-wide significance threshold (*P* < 5 × 10^−8^) and demonstrated independence under the linkage disequilibrium threshold of *r*^2^ < 0.001.^[15]^ The IVs necessitated SNPs with F-statistics >10 to mitigate the bias associated with weak IVs. The formula for calculating *F*-statistics is provided in Table S1, Supplemental Digital Content, https://links.lww.com/MD/Q849.^[16]^ SNPs with inconsistent alleles and palindromic SNPs with uncertain strands were either corrected or excluded during the data harmonization process.

### 2.4. Univariate Mendelian randomization analyses and sensitivity analyses

In our study, the 3 methods used in MR are inverse-variance weighting (IVW), MR-Egger, and weighted-median (WM). The IVW method serves as our primary analysis method, combining estimates from multiple genetic variants to provide a single causal estimate under the assumption that all IVs are valid.^[17]^ The MR-Egger method extends the IVW method by enabling the detection and correction of pleiotropy. It accounts for potential horizontal pleiotropy, where genetic variants may influence multiple traits.^[18]^ The WM method offers a robust estimate of the causal effect by calculating the median of the individual variant estimates, thereby minimizing the impact of any single variant on the result.^[19]^

Additionally, we employed several sensitivity analyses to assess the reliability of MR results, including Cochran *Q* test, the MR-Egger intercept, MR Pleiotropy Residual Sum and Outlier (MR-PRESSO), leave-one-out analysis, and funnel plots. Cochran *Q* test evaluates heterogeneity among the genetic variants used as IVs, with significant heterogeneity potentially indicating pleiotropy or violations of MR assumptions.^[20]^ If heterogeneity is detected and cannot be sufficiently explained or removed, we employed a random-effects IVW method. The MR-Egger intercept test detects directional pleiotropy, where a non-zero intercept suggests systematic effects of IVs on the outcome not mediated through the exposure.^[18]^ MR-PRESSO method identifies and corrects outliers among IVs that may cause pleiotropic effects, thereby enhancing the reliability of the causal estimate.^[21]^ Leave-one-out analysis sequentially excludes each IV to assess its influence on the overall causal estimate and identify potentially biasing outliers or influential variants.^[22]^ Funnel plots visually evaluate bias or heterogeneity among IVs, with asymmetry indicating potential pleiotropy or other biases.^[23]^ Furthermore, we applied False Discovery Rate (FDR) correction to adjust for multiple comparisons during simultaneous hypothesis testing using the same database.

### 2.5. Two step mediation Mendelian randomization analyses

**Figure 2.**
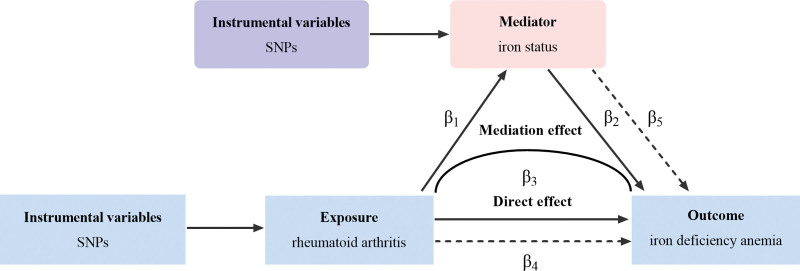
Framework for 2-step mediation analysis.

To explore the mechanisms through which an exposure might influence an outcome, a 2-step mediation MR method was employed using a 2-step process: The first step evaluates the relationship between the genetic instrument and the mediator. It quantifies how genetic predisposition to exposure is related to mediators that might lie on the pathway using UVMR. In the second step, the association between exposures and mediators on the outcome using MVMR.^[24]^ This step quantifies how the exposure and the mediator direct influenced by genetic predisposition to outcome. The mediation proportion is defined as β1×β4/(β1×β4+β5), where β1 represents the effect of exposure on the outcome using UVMR, and β4 and β5 represent the effects of exposure and mediator on the outcome using MVMR. β1×β4 represents the indirect effect of the mediator, while the β1×β4+β5 represents the total effect of the mediator.^[25]^ Moreover, the Delta method was used to calculate 95% confidence intervals. Figure 2 illustrates the process of 2-step MR.

## 3. Results

### 3.1. Genetic instruments variables for RA, SLE, SS, SSc and IDA

In the MR analysis, we identified 10 significant and independent SNPs associated with RA, 45 with SLE, and 5 with SS from genome-wide association studies (GWASs). However, the initial genome-wide significance threshold identified no SNPs for SSc. We subsequently relaxed this threshold to 5 × 10^-6^ and identified 7 SNPs for SSc. Detailed information on these SNPs is presented in Table S1, Supplemental Digital Content, https://links.lww.com/MD/Q849. The *F*-statistics of all SNPs ranged from 20.98 to 759.99. After harmonization with IDA, we removed palindromic and ambiguous SNPs and searched for proxy SNPs, ultimately identifying 10 SNPs for RA, 41 for SLE, 3 for SS, and 7 for SSc as IVs.

### 3.2. Mendelian randomization analysis for causal association of RA and IDA

**Figure 3.**
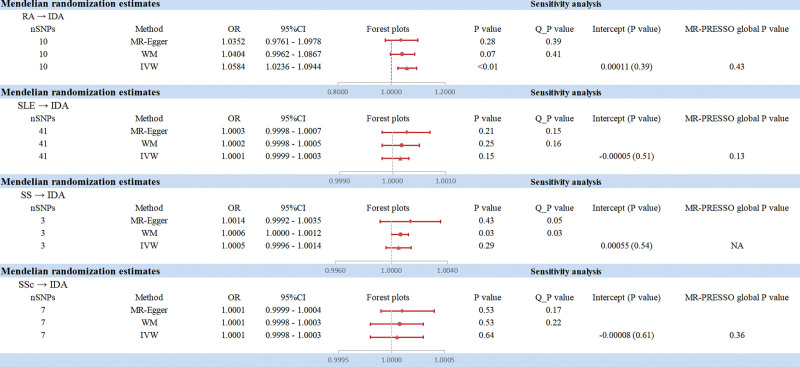
Estimates from MR analysis for RA, SLE, SS, SSc and IDA. IDA = iron-deficiency anemia, MR = Mendelian randomization, RA = rheumatoid arthritis, SLE = systemic lupus erythematosus, SS = Sjögren’s syndrome, SSc = systemic sclerosis.

**Figure 4.**
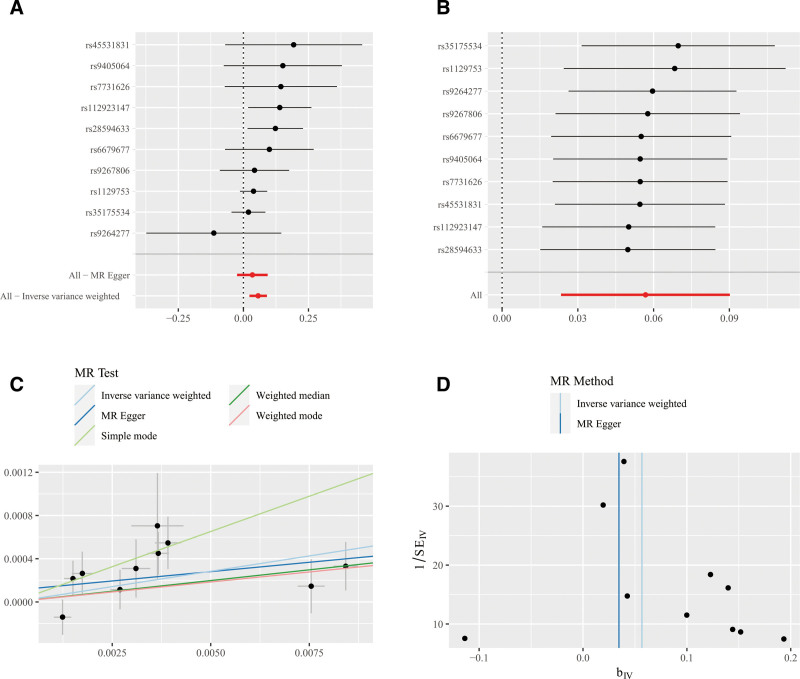
(A) MR estimates illustrate the relationship between RA and IDA, indicating a significant association (OR = 1.0584, 95% CI: 1.0236–1.0944, *P* <: ^$.01). (B) The leave-one-out test for RA and IDA suggests that our results are stable. (C) The Scatter plot of the association between RA and IDA. The MR-Egger intercept is 0.00011 (*P* = .39), suggesting that there is no significant horizontal pleiotropy in our study. Notably, the slopes across all 3 methods are directionally consistent, affirming the stability of our findings. (D) The funnel plot on RA and IDA. The funnel plots are symmetric, which shows that the absence of polymorphism. CI = confidence interval, IDA = iron-deficiency anemia, MR = Mendelian randomization, OR = odds ratio, RA = rheumatoid arthritis.

We found that genetically predicted RA was positively associated with IDA using the IVW method (OR = 1.058, 95% CI: 1.024–1.094, *P* <.01). However, no statistically significant association was observed between RA and IDA using the WM method or the MR-Egger method (Fig. 3). Sensitivity analyses, including the Cochrane Q test, MR-Egger intercept, MR-PRESSO global *P* value, leave-one-out analysis, and funnel plot, indicated no significant heterogeneity or horizontal pleiotropy, suggesting the stability and robustness of our results (Fig. 4). Given that more than 50% of the IVs in our study were valid and no significant horizontal pleiotropy was detected, we consider the IVW method to be robust. In the forward MR analysis, we found no significant association IDA on RA in the 3 method. Sensitivity analysis did not suggest significant heterogeneity and horizontal pleiotropy in our study (Fig. 3).

### 3.3. Mendelian randomization analysis for causal association of SLE, SS, SSc and IDA

No significant associations were observed between SLE, SSc and IDA using the IVW method (Figs. S1 and 3, Supplemental Digital Content, https://links.lww.com/MD/Q849). Given the significant heterogeneity identified between SS and IDA, a random-effects IVW method was employed, which indicated no significant association (Fig. S2, Supplemental Digital Content, https://links.lww.com/MD/Q849). Additionally, other sensitivity analyses revealed no significant heterogeneity or horizontal pleiotropy within this study (Fig. 3).

### 3.4. Two step mediation Mendelian randomization

To explore mediation effects between RA and IDA, we investigated the association between RA and iron status using the IVW method. Table 1 displays the MR estimates for the relationship between RA and various iron status indicators. A significant association was found between RA and ferritin levels after FDR correction (OR = 0.9966, 95% CI: 0.9938–0.9994, *P* = .01, P_FDR_ = 0.04). However, no significant associations were detected between RA and other iron status. Furthermore, we conducted MVMR analysis for both variables to explore the independent effects of RA and ferritin. The MVMR results indicate that RA and ferritin exert independent effects on IDA (RA: OR = 1.073, 95% CI: 1.005–1.146, *P* = .03; ferritin: OR = 0.997, 95% CI: 0.995–0.999, *P* < .01). We evaluated the mediation effect of RA on IDA through ferritin and found that the mediation effect was 1.16 × 10^−5^, and the mediated proportion was 1.63 × 10^−4^ (95% CI: 1.51 × 10^−4^–1.75 × 10^−4^).

**Table 1:** MR estimates of the associations from RA on iron status.

Iron status	OR	95% CI	*P*-value	*P*_FDR_ value
Iron	0.9988	0.9971–1.0005	.17	.24
Ferritin	0.9966	0.9938–0.9994	.01	.04
Transferrin	1.0008	0.9974–1.0043	.64	.64
Transferrin saturation	0.9991	0.9979–1.0004	.18	.24

CI = confidence interval, MR = Mendelian randomization, OR = odds ratio, RA = rheumatoid arthritis.

## 4. Discussion

In this study, we employed bidirectional UVMR and 2-step mediation MR analysis to investigate the genetic association between rheumatic diseases and IDA. Our findings indicate that RA may genetically increase the risk of IDA, with ferritin potentially acting as a mediator, suggesting that individuals with a genetic predisposition to RA are at an increased risk of developing IDA due to reduced ferritin levels. Within the power and design constraints of the present study, we found no evidence of a causal genetic relationship between IDA and SLE, SS, or SSc.

Anemia is a prevalent issue among individuals with RA, as confirmed by numerous studies. For example, Furst et al found that 16.7% of RA patients in a large U.S. cohort had low hemoglobin levels.^[26]^ Similarly, a German cohort study using the IQVIA Disease Analyzer database demonstrated a significant correlation between RA and anemia (OR = 1.32, 95% CI: 1.24–1.40).^[27]^ These findings underscore the importance of monitoring and managing anemia in RA patients to enhance their overall health outcomes.

The complexity of anemia in RA is further illustrated by its predominant forms: ACD and IDA. Interestingly, these 2 forms of anemia often coexist in the same individual, a condition known as “anemia of chronic disease (ACD) with iron deficiency or “mixed anemia.”^[4]^ A comprehensive analysis of 100 anemic RA patients revealed that approximately 20% had pure ACD, while a significant 80% exhibited both ACD and IDA.^[28]^ Moreover, Peeters et al found that about 40% of RA patients with anemia did not recover after iron supplementation, further indicating the prevalence of mixed anemia in this population.^[29]^

While the exact mechanisms linking RA to an increased risk of IDA remain unclear, several potential pathways have been proposed. Firstly, RA is associated with chronic inflammation, which can induce the production of hepcidin, a hormone synthesized by the liver that regulates iron homeostasis. Elevated hepcidin levels can inhibit iron release from macrophages and reduce intestinal iron absorption, resulting in iron-restricted erythropoiesis and anemia.^[30,31]^ Secondly, Nonsteroidal anti-inflammatory drugs (NSAIDs) are frequently used to alleviate pain and inflammation in RA patients. However, prolonged NSAIDs usage can lead to gastrointestinal side effects such as ulcers and bleeding, which can cause iron loss and contribute to IDA.^[32]^ Additionally, certain medications for RA, such as methotrexate and other disease-modifying antirheumatic drugs (DMARDs), may suppress bone marrow function, leading to decreased production of red blood cells and anemia.^[33]^ Thirdly, RA patients may experience reduced dietary iron intake or malabsorption due to gastrointestinal involvement or comorbidities, further contributing to iron deficiency.^[34]^

In contrast to RA, where there is a clear causal relationship IDA mediated by chronic inflammation and altered iron homeostasis, other rheumatic diseases such as SLE, SS and SSc exhibit distinct pathophysiological profiles that might not directly impact iron metabolism to the same extent. This difference can be attributed primarily to the variations in their immune response and the nature of tissue involvement. SLE is a multisystem autoimmune disease with a broad and complex immune response that can affect virtually any organ system. The immune dysfunction in SLE is characterized by autoantibody production, immune complex deposition, and complement activation, leading to widespread inflammation.^[35]^ However, the impact on iron metabolism in SLE is more often an indirect consequence of systemic inflammation rather than a direct interference with iron regulatory pathways.^[36]^ Moreover, the types of anemia observed in SLE are diverse, including hemolytic anemia from autoantibodies against red blood cells and ACD, which may overshadow the effects of iron deficiency alone.^[37]^ SS primarily targets exocrine glands, leading to symptoms like dry mouth and dry eyes due to lymphocytic infiltration. While SS can have systemic manifestations, the localized glandular involvement and the relatively less intense systemic inflammation imply that the mechanisms leading to anemia in SS are less likely to involve disrupted iron homeostasis as seen in RA. Instead, anemia in SS may result more from decreased erythropoiesis due to glandular dysfunction or the effects of chronic illness.^[38]^ SSc is characterized by progressive fibrosis affecting the skin and internal organs, along with vascular abnormalities. The pathogenesis of SSc revolves around 3 key elements: immune dysregulation, endothelial injury, and fibroblast activation leading to collagen deposition.^[39]^ These processes do not directly impact iron absorption or storage. Instead, anemia in SSc may be attributed to gastrointestinal malabsorption due to intestinal involvement or chronic renal impairment, rather than a primary disruption of iron metabolism.^[40]^

These distinctions in disease pathology and inflammatory patterns suggest that the mechanisms leading to IDA in RA are unique and not applicable to other rheumatic diseases. This understanding is crucial for developing disease-specific management strategies. In RA, addressing both the underlying inflammation and its impact on iron metabolism may potentially improve patient outcomes and reduce the burden of anemia. Further research is needed to explore detailed molecular pathways and to confirm these findings in more diverse populations. However, the challenge of distinguishing between IDA, ACD, or mixed anemia in patients with RA complicates the research. Most observational studies focus on anemia as the main outcome, resulting in a current lack of investigation into the causality of subtypes of anemia in this population. Our study is the first to demonstrate that the increased genetic risk of IDA in RA patients. The primary strength of our study lies in the employment of the MR design, which elucidates the causal relationship between RA and IDA, circumventing the inherent limitations associated with traditional observational studies. Furthermore, we used data from the most comprehensive collection of GWASs available to maximize the number of genetic instruments, thereby enhancing statistical power. Additionally, the predominance of European ancestry in all datasets minimizes the potential for demographic stratification bias.

We conducted a 2-step mediation MR analysis, demonstrating that RA partially increased the elevated risk of IDA through low ferritin levels. In the initial MR step, we established a causal relationship between RA and decreased ferritin levels. It is important to note that while ferritin levels are often elevated during acute episodes of RA, as ferritin is an acute-phase reactant that rises in response to inflammation,^[41]^ this elevation can potentially mask underlying iron deficiency and IDA, leading to misinterpretations of the true iron status in these patients. Importantly, the majority of RA patients included in this GWAS are in the chronic stage, where ferritin levels are typically reduced. This chronic stage data is crucial as it more accurately reflects the true iron status, avoiding the acute-phase elevation that can obscure iron deficiency. Our findings align with previous research,^[42]^ which observed decreased ferritin levels in chronic RA. Subsequently, the second MR step established that genetically predisposed low ferritin levels are associated with an increased risk of IDA, a finding consistent with many studies.^[43]^ In summary, these studies provide genetic evidence confirming the mediation effect of ferritin between RA and the risk of IDA, highlighting the importance of monitoring and managing ferritin levels in the RA population.

Several limitations should be acknowledged. Because an insufficient number of genome-wide significant instruments were available for the reverse MR analysis, we relaxed the significance threshold to 5 × 10^−6^, a step that may have weakened instrument strength and biased the causal estimates. We therefore calculated *F*-statistics and conducted extensive sensitivity tests to verify the robustness of these results. In addition, the small pool of instrumental SNPs reduces statistical power to detect moderate genetic effects. Autoimmune-related variants might also influence IDA through inflammation-mediated pathways unrelated to the primary exposures; such residual confounding cannot be fully ruled out by MR-Egger or MR-PRESSO. Moreover, the instruments employed may not capture relevant noncoding or epigenetic variation, and currently available GWAS data do not encompass the full regulatory landscape of rheumatic diseases. The lack of high-quality GWAS for other anemia subtypes prevented a broader analysis. Reliance on summary-level data precluded stratification by age, sex, and other demographic variables. Finally, all source cohorts were of European ancestry, limiting the generalisability of our findings to other populations.

## 5. Conclusion

In conclusion, this study employed large GWAS for MR analysis, aiming to explore causal relationships between rheumatic diseases and IDA. Our findings present strong genetic evidence of the association between RA and increased risk of IDA, and ferritin partially mediated this effect. These findings emphasize the importance of formulating prevention strategies and interventions for IDA in RA patients. To strengthen causal inference, future studies should integrate MR findings with phenome-wide association analyses and longitudinal cohorts that capture repeated iron-biomarker measurements, thereby enabling dynamic validation of the pathways proposed here.

## Acknowledgments

We express our gratitude to all the researchers who contributed to this MR study, and we appreciate the institutions and respective researchers who generously provided the data for this study. The MR analysis was executed using the 2-Sample MR (version 0.5.5) package in R (version 4.2.0).

## Author contributions

**Conceptualization**: Wei Huang.

**Data curation**: Wei Huang.

**Formal analysis**: Wei Huang, Tianyu Jin.

**Funding acquisition**: Yifan Cheng, Linyu Geng.

**Investigation**: Luyao Lv, Tianyu Jin, Yifan Cheng.

**Methodology**: Luyao Lv, Tianyu Jin.

**Project administration**: Tianyu Jin.

**Resources**: Tianyu Jin.

**Software**: Luyao Lv, Tianyu Jin.

**Supervision**: Tianyu Jin, Xuebing Feng.

**Validation**: Yifan Cheng, Xuebing Feng.

**Visualization**: Yifan Cheng.

**Writing – original draft**: Wei Huang, Yaqi Zhang, Linyu Geng, Xuebing Feng.

**Writing – review & editing**: Wei Huang, Luyao Lv, Yaqi Zhang, Tianyu Jin, Yifan Cheng, Linyu Geng, Xuebing Feng.

## Supplementary Material
