# Supplementary material for: Causality between rheumatic diseases and iron-deficiency anemia: A 2-sample, 2-step mediation Mendelian randomization investigation
Source: Medicine (Baltimore). 2026 May 12;104(49):e46345. doi: 10.1097/MD.0000000000046345 (PMC12688711; doi:10.1097/MD.0000000000046345)
Supplement: Supplementary file 1 [file medi-104-e46345-s001.pdf]

Supplementary Table 1. Characteristics of instrumental variables for RA, SLE, SS, and SSc.

	SNP	EA	OA	β	SE	P value	F-statistics
RA							
1	rs6679677	A	C	0.0031	0.0004	7.60E-18	73.38
2	rs7731626	A	G	-0.0015	0.0002	1.10E-11	45.69
3	rs112923147	G	A	0.0039	0.0003	7.50E-33	143.28
4	rs9264277	C	T	0.0012	0.0002	2.20E-08	31.04
5	rs45531831	T	C	0.0036	0.0007	3.60E-08	30.02
6	rs9267806	A	G	0.0027	0.0002	1.70E-27	118.48
7	rs28594633	A	G	0.0037	0.0003	4.70E-42	184.26
8	rs35175534	C	A	0.0075	0.0003	9.51E-108	486.84
9	rs1129753	T	C	0.0084	0.0003	3.20E-167	759.99
10	rs9405064	C	A	0.0017	0.0003	9.80E-11	41.68
SLE							
1	rs6679677	A	C	0.3365	0.0465	4.55E-13	52.39
2	rs4661543	G	T	0.2744	0.0424	9.40E-11	41.94
3	rs10912578	G	A	-0.2469	0.0310	1.65E-15	63.45
4	rs17849501	T	C	0.8109	0.0499	1.81E-59	264.48
5	rs6671847	A	G	0.1989	0.0290	6.64E-12	47.13
6	rs4916215	T	C	0.2231	0.0340	5.07E-11	43.15
7	rs12094036	C	T	-0.3285	0.0579	1.37E-08	32.24
8	rs13019891	T	G	-0.5621	0.0290	1.65E-83	374.85
9	rs2573219	C	A	0.5878	0.0429	1.13E-42	187.47

10	rs10200680	T	C	-0.2485	0.0425	4.96E-09	34.20
11	rs268124	T	C	0.1863	0.0324	8.60E-09	33.13
12	rs2459611	T	C	0.2614	0.0452	7.62E-09	33.37
13	rs4274624	T	C	-0.5596	0.0327	9.73E-66	293.25
14	rs10048743	T	G	-0.2311	0.0412	2.04E-08	31.46
15	rs34703115	C	T	-0.6162	0.1048	4.08E-09	34.58
16	rs1464446	T	G	-0.3285	0.0401	2.79E-16	66.94
17	rs9852014	G	A	0.6206	0.0493	2.26E-36	158.63
18	rs13136219	T	C	-0.1744	0.0278	3.50E-10	39.37
19	rs1078324	A	C	-0.7134	0.0782	7.11E-20	83.28
20	rs4388254	T	C	0.3784	0.0604	3.71E-10	39.26
21	rs2431697	C	T	-0.2231	0.0293	2.60E-14	58.02
22	rs6889239	C	T	0.2776	0.0317	2.19E-18	76.51
23	rs389884	G	A	0.9282	0.0432	2.92E-102	460.99
24	rs9274357	T	C	0.4574	0.0352	1.28E-38	168.91
25	rs7768653	T	C	-0.2070	0.0297	3.11E-12	48.62
26	rs12524498	T	G	-0.6733	0.1208	2.48E-08	31.07
27	rs58721818	T	C	0.6575	0.0756	3.38E-18	75.66
28	rs150180633	T	C	0.9282	0.0690	2.66E-41	181.19
29	rs28361029	A	G	-0.3857	0.0614	3.27E-10	39.50
30	rs35000415	T	C	0.5878	0.0415	1.86E-45	200.23
31	rs2736332	C	G	0.2776	0.0321	4.83E-18	74.95
32	rs7823055	T	G	-0.3507	0.0286	1.64E-34	150.11

33	rs7899626	T	C	0.1823	0.0333	4.19E-08	30.06
34	rs7097397	A	G	-0.1863	0.0287	8.60E-11	42.12
35	rs58688157	G	A	-0.2231	0.0336	2.97E-11	44.20
36	rs353608	G	A	0.1863	0.0280	2.93E-11	44.22
37	rs73050535	T	C	-0.7134	0.1241	9.11E-09	33.02
38	rs597808	G	A	-0.1625	0.0295	3.51E-08	30.40
39	rs1143679	A	G	0.5822	0.0400	5.03E-48	212.00
40	rs28834423	C	G	0.4574	0.0365	5.65E-36	156.80
41	rs13332649	G	A	-0.3147	0.0376	5.43E-17	70.17
42	rs143123127	A	G	0.4700	0.0840	2.23E-08	31.28
43	rs35251378	A	G	-0.2357	0.0324	3.61E-13	52.84
44	rs73068668	A	G	-0.3147	0.0575	4.40E-08	29.97
45	rs3747093	A	G	0.2624	0.0345	2.88E-14	57.81
SS							
1	rs35407265	G	A	-0.3687	0.0613	1.79E-09	36.18
2	rs2853986	C	T	0.7754	0.0742	1.43E-25	109.21
3	rs9265957	T	C	0.6963	0.0730	1.37E-21	90.98
4	rs3778754	G	C	0.2834	0.0407	3.16E-12	48.49
5	rs496315	C	T	0.2254	0.0408	3.21E-08	30.52
SSc							
1	rs2222631	A	G	0.3971	0.0831	1.78E-06	22.83
2	rs141520926	T	C	1.4345	0.3086	3.34E-06	21.61
3	rs72840563	C	G	0.9888	0.1965	4.88E-07	25.32

4	rs36030018	C	G	0.9239	0.1535	1.76E-09	36.23
5	rs3807307	C	T	0.3960	0.0833	1.96E-06	22.60
6	rs2501203	G	A	-0.4933	0.1077	4.63E-06	20.98
7	rs117642611	C	A	6.0160	1.3001	3.71E-06	21.41

RA, rheumatoid arthritis; SLE, systemic lupus erythematosus; SS, Sjogren's syndrome; SSc, systemic sclerosis; SNP, single nucleotide polymorphisms; EA, effect allele; OA, other allele; EAF, effect allele frequency; SE, standard error.

*F - statistic = β^2 / SE^2

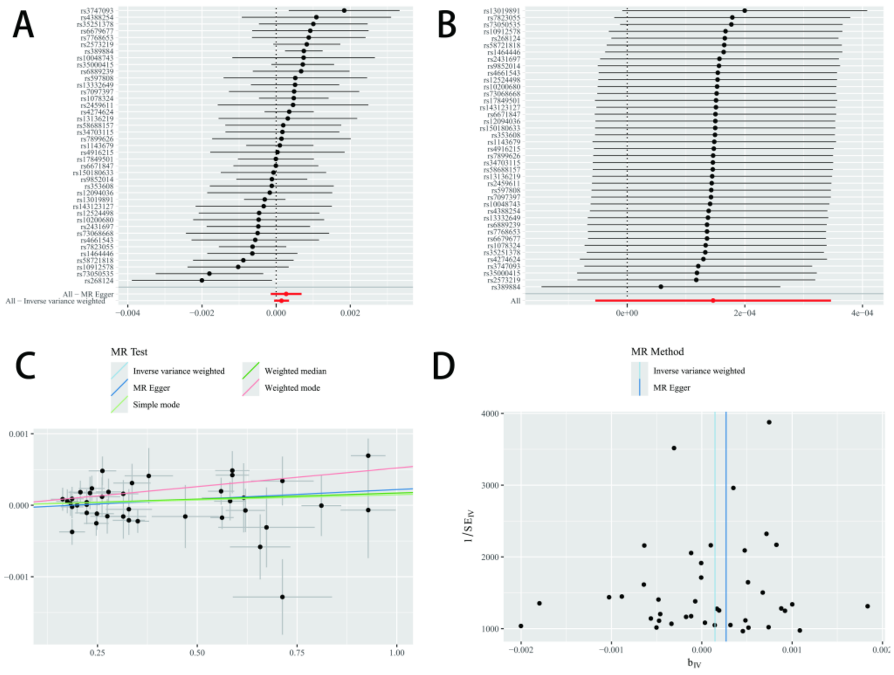

Supplementary Figure 1. A) MR estimates illustrate the relationship between SLE and IDA. This demonstrates no significant association between SLE and the risk of IDA. B) The leave-one-out plot for SLE and the risk of IDA highlights how causal estimates (represented as points with horizontal lines) change when individual SNPs are excluded. This test confirms the stability of our results. C) The scatter plot visualizing the association between SLE and IDA displays five lines, each representing different MR methods: IVW, MR-Egger, WM, weighted mode and simple mode. D) The funnel plot concerning SLE and IDA is symmetrical, signifying the absence of polymorphism.

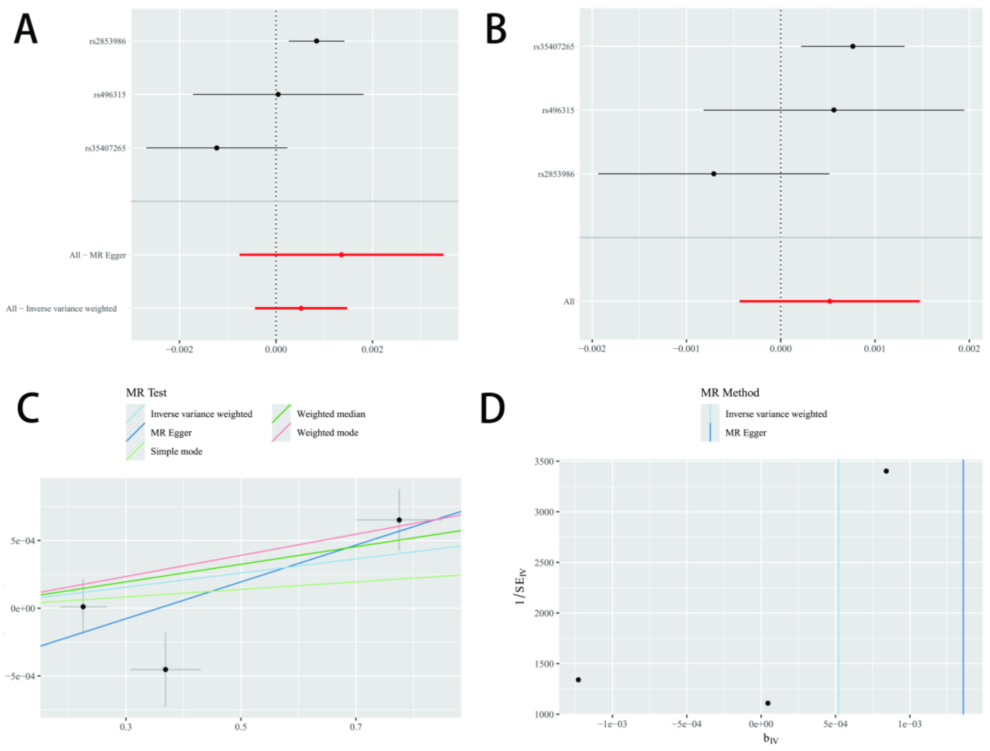

Supplementary Figure 2. A) MR estimates reveal no significant association between SS and IDA. B) The leave-one-out sensitivity analysis for SS and IDA is presented. C) A scatter plot details the association between SS and IDA. D) A funnel plot for SS and IDA demonstrates symmetry.

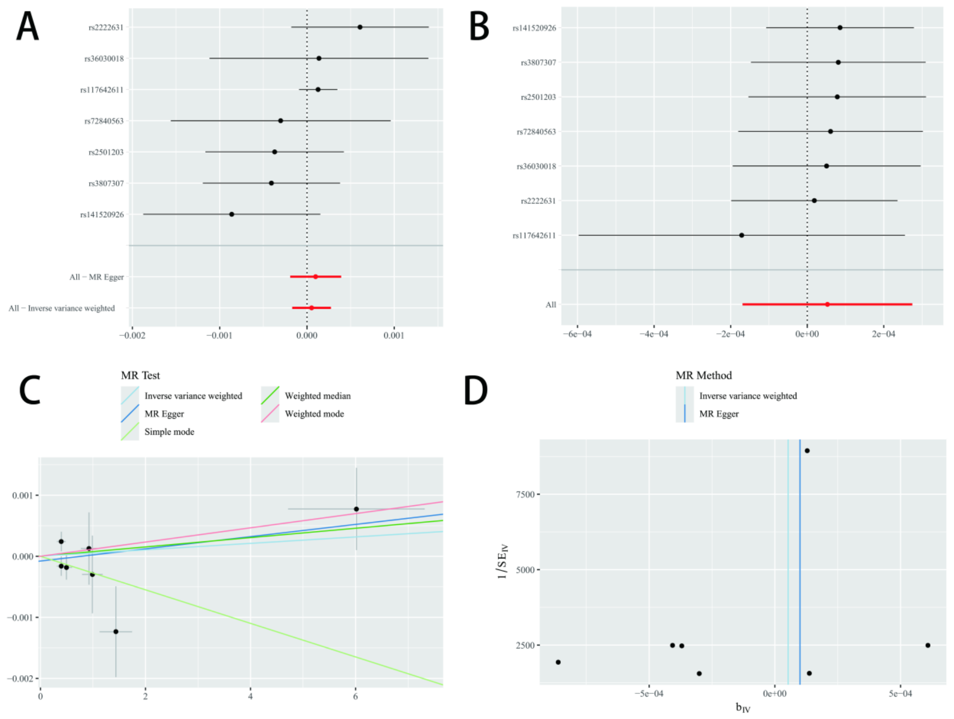

Supplementary Figure 3. A) MR estimates demonstrate the relationship between SSc and IDA. B) The leave-one-out plot concerning SSc and IDA. C) The scatter plot detailing the association between SSc and IDA. D) The funnel plot relating to SSc and IDA is symmetrical.
